# Multidrug-resistant enteroaggregative Escherichia coli associated with persistent diarrhea in Kenyan children.

**DOI:** 10.3201/eid0303.970317

**Published:** 1997

**Authors:** W K Sang, J O Oundo, J K Mwituria, P G Waiyaki, M Yoh, T Iida, T Honda

**Affiliations:** Kenya Medical Research Institute, Nairobi, Kenya.

## Abstract

To study the association of multidrug-resistant enteroaggregative Escherichia coli with persistent diarrhea in Kenyan children, stool specimens were obtained from 862 outpatients under 5 years of age from July 1991 to June 1993. E. coli O44 was identified as the sole bacterial pathogen in four patients experiencing at least 14 days of fever, vomiting, and diarrhea. Disk diffusion testing showed E. coli O44 resistance to tetracycline, ampicillin, erythromycin, trimethoprim-sulphamethoxazole, and amoxicillin/clavulanate and sensitivity to chloramphenicol, nalidixic acid, azithromycin, and cefuroxime. Further studies are needed to clarify the epidemiology, clinical spectrum, and pathogenesis of enteroaggregative E. coli infection.


### Multidrug-Resistant Enteroaggregative *Escherichia coli* Associated with Persistent Diarrhea in Kenyan Children

To study the association of multidrug-resistant enteroaggregative *Escherichia coli* with persistent diarrhea in Kenyan children, stool specimens were obtained from 862 outpatients under 5 years of age from July 1991 to June 1993. *E. coli* O44 was identified as the sole bacterial pathogen in four patients experiencing at least 14 days of fever, vomiting, and diarrhea. Disk diffusion testing showed *E. coli* O44 resistance to tetracycline, ampicillin, erythromycin, trimethoprim-sulphamethoxazole, and amoxicillin/clavulanate and sensitivity to chloramphenicol, nalidixic acid, azithromycin, and cefuroxime. Further studies are needed to clarify the epidemiology, clinical spectrum, and pathogenesis of enteroaggregative *E. coli* infection.

*Escherichia coli* infection is an important cause of illness and death in infants in developing countries (1). On the basis of patterns of adherence to tissue culture cells (HEp-2 or HeLa), *E. coli* strains can be classified into three groups: localized, diffuse, and aggregative (2). Much remains unknown about these strains. Enteroaggregative *E. coli* (EAggEC), which exhibits aggregative adherence, has been associated with diarrhea in children in Chile (3) and with persistent diarrhea in children in India (4). We report the first evidence of multidrug-resistant EAggEC associated with persistent diarrhea in Kenyan children.

From July 1991 to June 1993, stool specimens from 862 outpatients under 5 years of age at Malindi Hospital were examined for pathogenic organisms. Standard methods for isolating enteric pathogens were used. Laboratory tests to detect pathogenic factors, e.g., verotoxins (VT) in cultures of all *E. coli* isolates, were done by applying the conventional tissue culture method (which uses the Vero cell line [5] and the VT1 or VT2 genes [6]) and polymerase chain reaction (7). The genes for heat-labile enterotoxin and heat-stable enterotoxin, VT, and invasiveness were tested by DNA probes on all *E. coli* strains. The strains were further examined for adherence to HEp-2 cells (8) and tested by the disk diffusion method (9) for susceptibility to the antibiotics chloramphenicol, erythromycin, ampicillin, nalidixic acid, cefuroxime, trimethoprimsulphamethoxazole, amoxicillin/clavulanate, tetracycline, and azithromycin.

Bacterial pathogens were found in 27.7% of the samples; 119 *E. coli* isolates were obtained. The results indicated that many of the bacteria, e.g., pathogenic *E. coli*, *Salmonella* spp., and *Shigella* spp. (Table), had been transmitted by the fecal-oral route. *E. coli* O44 was isolated from four patients; the isolates occurred in an aggregative adherence pattern as chains and nearly random aggregates on HEp-2 cells.

The case descriptions of the four patients from whom the O44 strains were isolated are as

Table. Identification of enteric pathogens in children with diarrhea.

| Pathogens (number tested) | Number | Percentage % |
|---|---|---|
| Bacteria (862) | 239 | 28.0 |
| *Escherichia coli* | | |
| enteropathogenic *E. coli* (EPEC) | 71 | 8.0 |
| EAgg *E. coli (ETEC)* | 4 | 0.5 |
| enterotoxigenic *E. coli* | 43 | 5.0 |
| enterohemorrhagic *E. coli* | 1 | 0.1 |
| *Salmonella* spp. | 63 | 7.3 |
| *Shigella* spp. | 56 | 6.5 |
| *Campylobacter* spp. | 42 | 4.9 |
| *Vibrio parahaemolyticus* | 4 | 0.5 |
| Parasites (862) | 109 | 12.6 |
| *Entamoeba histolytica* | 67 | 7.8 |
| *Giardia lamblia* | 42 | 4.9 |
| Viruses (427) | | |
| Rotavirus | 69 | 16.2 |

Mixed infections: ETEC/EPEC = 12, EPEC/*Campylobacter* = 8, *Salmonella*/ETEC = 7, *Salmonella*/EPEC = 3, *Shigella*/*Campylobacter* = 2, *Vibrio*/*Shigella* = 2, *Shigella*/*Salmonella* = 1, EPEC /*Shigella* = 6, ETEC/*Shigella* = 4.

follows: Patient 1, age 28 months, had fever, gross blood in stool, vomiting, and diarrhea lasting 14 days; Patient 2, age 35 months, had fever, abdominal pain, nausea, vomiting, and diarrhea lasting 15 days; Patient 3, age 24 months, had fever, gross blood in stool, vomiting, and diarrhea lasting 14 days; and Patient 4, age 26 months, had fever, abdominal pain, vomiting, and diarrhea lasting 14 days. The patients were not related and lived in different communities. These particular strains of *E. coli* O44 had similar patterns of resistance to tetracycline, ampicillin, erythromycin, trimethoprimsulphamethoxazole, and amoxicillin/clavulanate; they were all sensitive to chloramphenicol, nalidixic acid, azithromycin, and cefuroxime.

Persistent diarrhea is increasingly recognized as an important public health problem among children in developing countries (10) and is a research priority of the Diarrhoeal Diseases Control Programme of the World Health Organization (11). In the four patients from whom it was isolated, EAggEC was the sole bacterial pathogen recovered. However, tests for parasitic causes of persistent diarrhea, such as *Cyclospora* and *Cryptosporidium*, were not available at the time of our study and were not performed. The association of EAggEC with persistent diarrhea will be strengthened by extending this kind of study to other areas in Kenya and identifying all causes of persistent diarrhea.

Tetracycline**,** ampillicin, and trimethoprimsulphamethoxazole are recommended by the Kenyan Ministry of Health for the empiric treatment of diarrhea. These drugs were largely ineffective against *Shigella* spp. and EAggEC. Our results are consistent with the findings of Yamamoto et al., who found multidrug resistance in EAggEC strains from Thailand, Mexico, Chile, and Peru (12), and suggest that monitoring sensitivity to antibiotics in Kenya is necessary for optimum selection of effective antibiotics and elimination of antibiotics with little therapeutic value. Similarly, the epidemiology, clinical spectrum, and pathogenesis of EAggEC infection and the reservoir of the putative etiologic agent are still poorly understood or unknown. Further clinical, epidemiologic, and laboratory studies are needed to clarify these issues.

### Acknowledgments

We are grateful to Dr. B.L. Smoak, U.S. Army Medical Research Unit/Kenya Medical Research Institute, for reviewing the paper. This work was supported by the Japanese International Cooperation Agency (JICA) and the Kenya Medical Research Institute.

**W.K. Sang,* J.O. Oundo,* J.K. Mwituria,* P.G. Waiyaki,* M. Yoh,† T. Iida,† and T. Honda†**
*Kenya Medical Research Institute, Nairobi, Kenya; and †Osaka University, Osaka, Japan

### References

1. Levine MM. *Escherichia coli* that cause diarrhea: enterotoxigenic, enteropathogenic, enteroinvasive, enterohemorrhagic and enteroadherent. J Infect Dis 1987;15:377-89.
2. Scaletsky ICA, Silva MLM, Trabulsi LR. Distinctive patterns of adherence of enteropathogenic *Escherichia coli* to HeLa cells. Infect Immun 1984;45:534-6.
3. Nataro JP, Kaper JB. Patterns of adherence of diarrhaegenic *Escherichia coli* to HEp-2 cells. Pediatr Infec Dis J 1987;6:829-31.
4. Bhan MK, Raj P, Levine MM. Enteroaggregative *Escherichia coli* association with persistent diarrhea in a cohort of rural children in India. J Infect Dis 1989;159:1061-4.
5. Konowalchuk J, Speirs JI, Stavric S. Vero response to a cytotoxin of *Escherichia coli*. Infect Immun 1977;18:775-9.
6. Tomatsukuri S, Yamamoto K, Shibata S, Leaneo F, Honda T. Detection of a heat-labile enterotoxin gene in enterotoxigenic *Escherichia coli* by densitometric evaluation using highly specific enzyme-linked oligonucleotide probes. Eur J Clin Microbiol Infect Dis 1991;10:1048-55.
7. Pollard DR, Johnson WM, Lior H, Tyler SD, Rozee KR. Rapid and specific detection of verotoxin genes in *Escherichia coli* by the polymerase chain reaction. J Clin Microbiol 1990;28:540-5.
8. Cravioto AR, Gross J, Scotland SM, et al. An adhesive factor found in strains of *Escherichia coli* belonging to the traditional infantile enteropathogenic serotype. Current Microbiology 1979;3:95-9.
9. Bauer AW, Kirby WMM, Sherris JC, Turk M. Antibiotic susceptibility testing by a standardized single disc method. Am J Clin Pathol 1966;4:493-6.
10. Bhan MK, Arora NK, Ghai OP, Ramachandran K. Major factors in diarrhoea related mortality among rural children. Indian J Med Res 1986;83:9-12.
11. World Health Organization: Diarrhoeal Disease Control Programme: persistent diarrhoea in children—research priorities. Geneva: World Health Organization; 1985; CDD/DDM/85.1.
12. Yamamoto T, Echeverria P, Yokota T. Drug resistance and adherence to human intestines of enteroaggregative *Escherichia coli*. J Infect Dis 1992;165:744-9.
